# High Solubility and Bioavailability of Lobster Shell-Derived Calcium for Significantly Proliferating Bone and Skin Cells In Vitro

**DOI:** 10.3390/md21060358

**Published:** 2023-06-11

**Authors:** Trung T. Nguyen, Thanh Hoang, Tuyet Pham, Vi Khanh Truong, Xuan Luo, Jian Qin, Wei Zhang

**Affiliations:** 1 College of Science and Engineering, Flinders University, Adelaide, SA 5042, Australia; thanh.hoanghai@flinders.edu.au (T.H.); jian.qin@flinders.edu.au (J.Q.); 2 Advanced Marine Biomanufacturing Laboratory, Centre for Marine Bioproduct Development, College of Medicine and Public Health, Flinders University, Adelaide, SA 5042, Australia; 3 Biomedical Nanoengineering Laboratory, College of Medicine and Public Health, Flinders University, Adelaide, SA 5042, Australia; pham0261@flinders.edu.au (T.P.); vikhanh.truong@flinders.edu.au (V.K.T.); 4 Flinders Institute for Nanoscale Science and Technology, College of Science and Engineering, Flinders University, Adelaide, SA 5042, Australia; xuan.luo@flinders.edu.au; 5 Marine Bioproducts Cooperative Research Centre, Adelaide, SA 5042, Australia

**Keywords:** lobster shells, multi-product biorefinery, value-added products, microwave-intensified extraction, lobster mineral, nutraceutical calcium, wound healing, bone health

## Abstract

Shell wastes pose environmental and financial burdens to the shellfish industry. Utilizing these undervalued shells for commercial chitin production could minimize their adverse impacts while maximizing economic value. Shell chitin conventionally produced through harsh chemical processes is environmentally unfriendly and infeasible for recovering compatible proteins and minerals for value-added products. However, we recently developed a microwave-intensified biorefinery that efficiently produced chitin, proteins/peptides, and minerals from lobster shells. Lobster minerals have a calcium-rich composition and biologically originated calcium is more biofunctional for use as a functional, dietary, or nutraceutical ingredient in many commercial products. This has suggested a further investigation of lobster minerals for commercial applications. In this study, the nutritional attributes, functional properties, nutraceutical effects, and cytotoxicity of lobster minerals were analyzed using in vitro simulated gastrointestinal digestion combined with growing bone (MG-63), skin (HaCaT), and macrophage (THP-1) cells. The calcium from the lobster minerals was found to be comparable to that of a commercial calcium supplement (CCS, 139 vs. 148 mg/g). In addition, beef incorporated with lobster minerals (2%, *w*/*w*) retained water better than that of casein and commercial calcium lactate (CCL, 21.1 vs. 15.1 and 13.3%), and the lobster mineral had a considerably higher oil binding capacity than its rivals (casein and CCL, 2.5 vs. 1.5 and 1.0 mL/g). Notably, the lobster mineral and its calcium were far more soluble than the CCS (98.4 vs. 18.6% for the products and 64.0 vs. 8.5% for their calcium) while the in vitro bioavailability of lobster calcium was 5.9-fold higher compared to that of the commercial product (11.95 vs. 1.99%). Furthermore, supplementing lobster minerals in media at ratios of 15%, 25%, and 35% (*v*/*v*) when growing cells did not induce any detectable changes in cell morphology and apoptosis. However, it had significant effects on cell growth and proliferation. The responses of cells after three days of culture supplemented with the lobster minerals, compared to the CCS supplementation, were significantly better with the bone cells (MG-63) and competitively quick with the skin cells (HaCaT). The cell growth reached 49.9–61.6% for the MG-63 and 42.9–53.4% for the HaCaT. Furthermore, the MG-63 and HaCaT cells proliferated considerably after seven days of incubation, reaching 100.3% for MG-63 and 115.9% for HaCaT with a lobster mineral supplementation of 15%. Macrophages (THP-1 cells) treated for 24 h with lobster minerals at concentrations of 1.24–2.89 mg/mL had no detectable changes in cell morphology while their viability was over 82.2%, far above the cytotoxicity threshold (<70%). All these results indicate that lobster minerals could be used as a source of functional or nutraceutical calcium for commercial products.

## 1. Introduction

Global aquaculture and caught fish reached 177.8 million tons in 2020, with 16 million tons contributed from crustacean species [1]. The commercial processing of fish and crustaceans annually produces about 81 million tons of byproducts (8 million tons of crustaceans and 10 million tons of bivalve shellfish [2]), of which 32 million tons are discarded as biowaste [3,4]. The waste from lobster species globally in 2012 was estimated to be about 50,000 tons, dominated by shell wastes (shells, the carapace, heads, and legs) [5]. Discarding fish and lobster biowaste in landfills or dumping them into the seas causes air and water pollution. Managing and treating these biowastes could cost up to AUD 150 per ton in Australia and developed countries. Continuously producing and discarding these fish and lobster processing byproducts without developing efficient processes for complete utilization would therefore lead to disposal issues with high treatment costs and severe pollution. Since crustacean and lobster shells have been identified as important sources of minerals (36% of dry lobster shells), proteins (29%), and commercial chitin or chitin derivatives (23–25%), with numerous applications in different industries such as environmental, agricultural, food, pharmaceutical, biomedical, and healthcare [5,6,7], they have been utilized primarily for the commercial production of chitin. Wholly and efficiently utilizing these shells would dramatically minimize their environmental impact while adding more value to maximize their economic profitability.

The commercial production of crustacean shell chitin, mainly through chemical processes, is environmentally unfriendly and generates secondary products. The chitin in lobster shells is tightly associated with proteins and pigments. This is reinforced by calcification to make a strong biological matrix. To produce high-quality chitin for biological applications, such as biomedicine, these impurities should be removed entirely. Conventionally, shells are deproteinized with strong alkalis (NaOH or KOH) at various concentrations (0.125–5.0 M) and temperatures up to 160 °C from a few minutes up to a few days. During this process, corrosive inorganic acids (HNO_3_, H_2_SO_4_, and H_3_PO_4_ but preferably HCl at 0.5–11.0 M, −20–70 °C, and 0.5–24 h) and oxidative reagents (acetone, NaClO, and H_2_O_2_) are used for demineralization and depigmentation, respectively [8,9]. Using these strong, hazardous, and corrosive chemicals requires costly corrosive-resistant equipment and devices, and harms operating workers and the environment. Notably, it requires a large volume of chemicals or water for either additional neutralizing or intensively washing products to remove these chemical residues. This yields a massive quantity of harmful effluent water that is costly to treat. Moreover, harsh and thermal treatments with concentrated alkaline, acidic, and oxidative chemicals can lead to partial deacetylation, depolymerization of chitin, or/and racemizations, resulting in inconsistent physiological properties of the final product. Moreover, these methods cannot recover compatible proteins, pigments, and minerals for other value-added products.

The microwave-intensified biorefinery of lobster shells advantageously facilitated the recovery of multiple products with favorable functional properties and nutritional profiles [10,11,12,13,14]. Microwave-assisted extraction (MAE) has recently emerged as a promising technology for efficiently extracting different functional ingredients and bioactive compounds with a higher yield, lower solvent usage, and shorter extraction time [10]. The pretreatment of biomass with microwaves was reported to produce final products with higher bioactivities and better functionalities [11]. Additionally, microwave energy could improve the rates of enzymatic processes (the hydrolysis of starch, protein, and chitin) [12,13,14] or chemical reactions (demineralization of shrimp shells) [15,16,17]. Therefore, this has been innovatively applied in the enzymatic deproteinization and lactic-acid demineralization of lobster shells for multi-product biorefinery, including chitin, protein, and minerals. Thus, these lobster shell-derived products are commercially valuable and produce high yields with good quality [18]. Lobster shell protein has also been found to be highly digestible with commercially attractive functionalities [19], while lobster chitin has exhibited excellent physicochemical properties for various applications. Importantly, lobster minerals also had a favorable nutritional composition with calcium enrichment [20].

The high demand for calcium in human nutrition highlights the potential of lobster minerals as an alternative source of functional, dietary, or nutraceutical calcium. Calcium is an essential macro-mineral for the structural integrity of teeth and the strength of bones, besides playing vital roles in regulating the critical functions of nerve impulses, muscle contractions, and the activities of various enzymes [21,22]. Calcium deficiency has also been linked to specific chronic diseases such as osteoporosis, fluorosis, hypertension, cancer, and reduced blood clotting ability [23]. The required average intake of calcium is about 900 mg/day for adults and 1200 mg/day for both adolescents and older people, as suggested by FAO/WHO 2001. The intake of calcium via diet is essential for human health in general, particularly for those in a period of maximum growth, such as childhood, adolescence, or during lactation [24,25]. Although milk and dairy products are known as the most common and trusted sources for calcium intake, many Asian people do not drink milk due to lactose indigestion and intolerance, which makes them intolerant to milk and dairy products [26]. Seafood and seafood products often serve as alternatives for dietary calcium supplements. The regular intake of over 250 g of seafood per week has been associated with a greater bone mineral density [27] and the intake of small fish with bones increased the calcium bioavailability in rats. In addition, bony fish have been considered an essential source of dietary calcium, especially for those with low Intakes of milk and dairy products [28]. Therefore, fish bones and crustacean shells have been identified as alternative sources of dietary calcium for various applications [26,29]. Indeed, the fish bones and crab shells included in aquafeeds as calcium supplements for Atlantic cods were found to positively affect their weight gain [30]. Recently, fish bones have been utilized as a highly bioavailable calcium source for growing pigs; piglets fed diets formulated with fish bones had significantly higher calcium absorption than that of the controls [31]. This positive result was also observed in young healthy men with calcium generated from salmon and cod bones [32]. Therefore, calcium derived from bone meals and shells has been utilized in food processing [26] and nutraceutical supplements [33]. Advantageously, this alternative calcium was found to be much more absorbable than traditional calcium supplements [34]. This is particularly significant for calcium-rich organic salts such as calcium lactate [35]. Although it has been used for calcium deficiency treatment [36] and calcium is known to have vital functions in bone health [37] and wound healing [38], this has not yet been evaluated in regard to lobster minerals. Therefore, this study aims to evaluate the functional attributes, nutritional values, and nutraceutical applications of calcium-rich lobster minerals.

## 2. Results

### 2.1. Lobster Minerals as a Calcium-Rich Source for Various Applications

**Figure 1:**
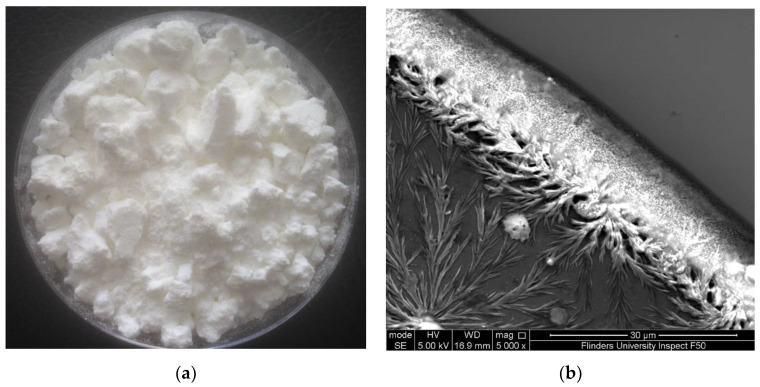
(**a**) Lobster mineral produced by the microwave-intensified lactic-acid demineralization of lobster shell [20] and (**b**) crystal structure of lobster mineral using scanning electron microscopy (SEM).

The solution generated from microwave-intensified lactic-acid demineralization of lobster shells was spray-dried to obtain white powder (Figure 1a). This product is referred to as lobster mineral because it was reported to contain very low contents of lipids and protein (0.4% and 1.3%, respectively) but a high content of minerals (88% of the dry weight) [39]. As reported in our previous research on the lobster mineral profile [20], the contents of magnesium, phosphorus, sodium, and other elements in the lobster mineral were, respectively, 6%, 4%, 2%, and 1%. They were much lower than that of calcium, which accounted for 87%, with a crystal structure identified as lactate calcium (Figure 1b). Such a favorable nutritional profile of the lobster minerals coupled with its high mineral content, which compares to that of tuna bone powder produced via alkaline digestion (77.9%) [40], makes it promising for commercial marketing as a source of nutritional minerals.

To further evaluate lobster mineral as a valuable source of dietary or nutraceutical calcium, the level of this mineral nutrient in the product (in the form of lactate calcium as the main ingredient) was determined and compared to that of a commercially available calcium supplement (with calcium carbonate as the major component). Although lactate calcium has been known to contain only 13% of elemental calcium compared to 40% of calcium carbonate [36], the calcium level of the lobster mineral was found to be 139 mg/g, comparable to that of the commercial rival (148 mg/g). This highlights the promising application of lobster minerals as a calcium-rich source for foods, calcium-fortified products, and nutraceutical supplements.

### 2.2. Lobster Mineral as a Functional Ingredient or Nutrient in Foods and Calcium-Fortified Products

#### 2.2.1. Water Holding and Oil Binding Capacity of Lobster Mineral

**Figure 2:**
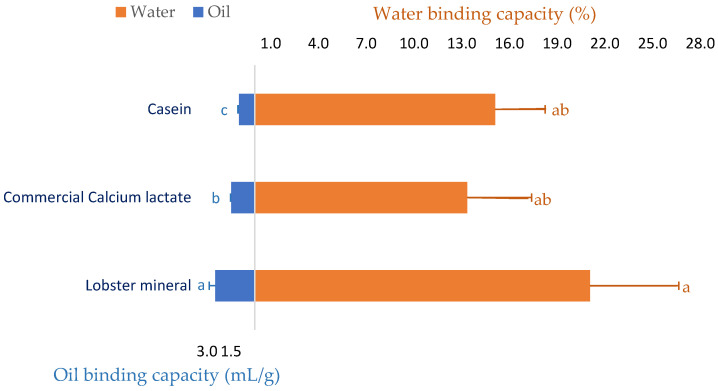
Oil binding and water holding capacity of beef mince supplemented with lobster mineral compared to commercial calcium lactate and casein. The results are shown as the mean of triplicates with standard deviations. Plots with the same letters are statistically insignificant (*p* > 0.05).

Since lobster mineral has been demonstrated to enrich calcium similar to its commercial rival product (CCS) and calcium has exhibited several beneficial functionalities in meat [41] and food processing [42,43], the water and oil binding capacity of the calcium-rich lobster mineral was investigated. As seen in Figure 2, beef mince incorporated with commercial calcium lactate (CCL) at a ratio of 2% (*w*/*w*) improved the water binding capacity of the meat product by 13.3%. Formulating calcium-rich lobster minerals at the same ratio in beef produced a more noticeable result. Over 21.1% of the initial amount of added water was retained in the lobster-mineral-formulated beef mince. This result is consistent with a previous study that reported that pork sausages supplemented with an oyster calcium powder (0.3 or 0.5%) resulted in meat products with a significantly higher water retaining capacity, hardness, and chewiness [44]. The positive effects of calcium mixtures on the quality of meat products were comparable to those of synthetic phosphate [41]. Thus, various calcium powders have been investigated as a replacement for synthetic phosphate on the quality properties of ground pork meat products [45]. Oyster shell calcium combined with eggshell calcium, at respective ratios of 0.2 and 0.3%, was proposed to replace synthetic phosphate in pork products [46]. Moreover, the water binding capacity of the lobster-mineral-incorporated product was also compared to the beef mince incorporated with casein, a milk protein commonly added to food products, to improve their capacity to bind water and oil [47,48]. Although the water retention of the two products was comparable (21.1 vs. 15.1%), the oil binding capacity of the lobster mineral was appreciably higher than that of its rivals (2.5 vs. 1.5 mL/g of CCL and 1.0 mL/g of casein, Figure 2). Such significant results demonstrated that lobster mineral may be used as a functional ingredient in meat products since supplementing calcium lactate at 0.2–0.4% to cured beef sausages was reported to achieve a more significant redness, oxidative stability, and softer texture. Notably, using calcium lactate for co-extruding sausages coated with alginate obtained high efficiency because calcium interacts well with alginate to form the casing during the co-extrusion of the sausages [49].

#### 2.2.2. Higher Solubility of Lobster Mineral Is Promising for Calcium-Fortified Beverages

**Figure 3:**
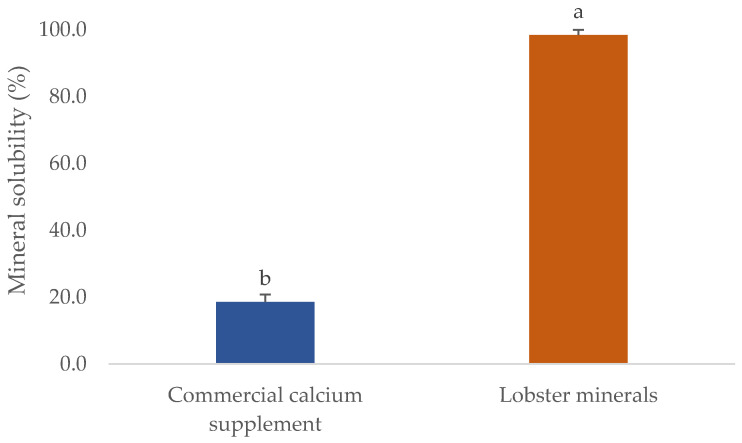
Solubility of lobster mineral compared with a commercial calcium complex supplement. The results are shown as the mean of triplicates with standard deviations. Plots with the same letters are statistically insignificant (*p* > 0.05).

One of the critical parameters in which the quality of mineral extracts scored is their solubility. This will significantly influence their commercial applications, particularly for processing foods, calcium-fortified products, or nutraceutical supplements. The solubility of several commercial fish bone minerals was found to reach the highest level at pH 3, ranging from 33 to 50%. However, their solubility in water or at pH 7 was very low, even when the process was carried out at 50 °C (solubility was lower than 10%) [50]. This was also observed in the CCS used in this study (Figure 3). The CCS had a solubility in water of 18.6%, five-fold lower than that of lobster minerals (98.4% at pH 7.5). The different forms of calcium salts could explain this. The lobster mineral contained calcium lactate as the main ingredient, whereas CCS primarily contained calcium carbonate. The solubility of organic calcium salts, including calcium lactate, was reported to be much higher than that of its inorganic forms [35]. Even the hydroxyapatite lactate produced from fish bones with lactic acid was much more soluble than the products derived from other organic acids (citric and malic) [51]. Notably, the higher solubility of calcium lactate was found not only in the minerals derived from lobster shells but also in those from mussel shells [52]. This result demonstrates that the lobster mineral has excellent potential in commercial products requiring high solubilities, such as calcium-fortified beverages and supplements.

### 2.3. High Solubility of Gastric-Digested Lobster Calcium Paired with Its In Vitro Bioavailability Makes Lobster Mineral Favorable for a Dietary or Nutraceutical Calcium Source

**Figure 4:**
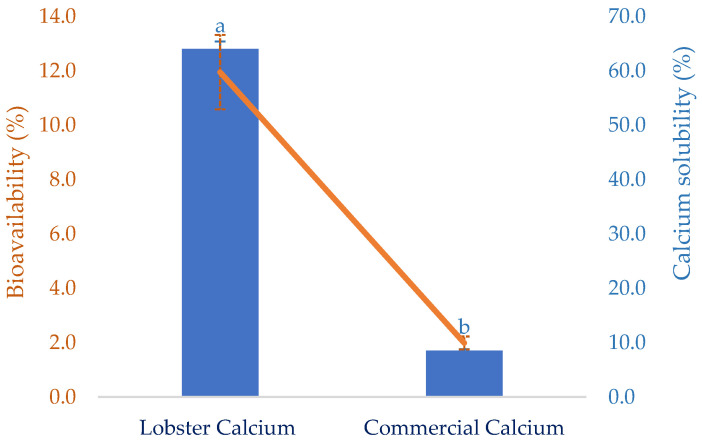
Solubility and in vitro bioavailability of lobster calcium compared to a commercial calcium product. The results are shown as the mean of triplicates with standard deviations. Plots with the same letters are statistically insignificant (*p* > 0.05).

The solubility of calcium in the gastrointestinal tract is critically vital because it decides the quantity of calcium that can be absorbed. However, certain dietary factors can influence the calcium-solubilizing process. This certainly affects the calcium bioavailability at the absorptive surface of intestinal cells. Thus, this attribute of the lobster mineral, after simulated gastric digestion, was determined as seen in Figure 4. Compared to calcium derived from a commercial supplement, the solubility of lobster calcium was over seven-fold higher than that of the commercial rival (64.0 vs. 8.5%). This could be a result of the exceptionally high solubility of the lobster mineral compared to the calcium complex combined with the biochemical advantages of calcium lactate versus calcium carbonate [35]. Therefore, the high solubility of lobster minerals is a great nutritional indicator for the product to be marketed as an excellent dietary or supplemental calcium source since there is a positive relationship between calcium solubility and its bioavailability [21].

Bioavailability has been used as an essential nutritional indicator to reflect the nutritional values or potential health effects of various biomolecules or bioproducts. It is defined as the degree to which the amount of an ingested nutrient is absorbed and available to the body. This was favorably demonstrated for lobster calcium using simulated gastrointestinal digestion, as shown in Figure 4. Compared to calcium derived from cow’s milk, with a bioavailability of 17 ± 0.8%, as reported in another study [53], the value of lobster calcium was relatively low (11.95 ± 0.22%). However, lobster calcium was much more bioavailable than the commercial calcium supplement. The former had a six-fold higher bioavailability than the latter (11.95 vs. 1.99%). This could be due to the superior solubility of both lobster mineral and calcium. Additionally, the nutritional advantage of calcium lactate over calcium carbonate may be another favorable contributor. Moreover, calcium lactate produced from mussel shells, instead of lobster shells, was also shown to be highly bioavailable [52]. One study supported this by comparing the bioavailability of calcium lactate with calcium carbonate by measuring the rise in serum calcium in 20 healthy young men for two hours after ingesting 500 mg of two calcium salts. The serum calcium was observed to increase by 7.6% in the subjects who ingested calcium lactate, while the increase for those who consumed calcium carbonate was about 5.5% [35]. Moreover, measuring serum calcium after oral supplementation at 0 and 24 h(s) was also performed for 12 postmenopausal females aged between 55 and 75 years old to compare the bioavailability of calcium carbonate (conventional calcium supplement) to that of fish bone calcium [34]. Significantly higher serum calcium was obtained for the alternative calcium, which was then suggested as a nutraceutical supplement for treating postmenopausal osteoporosis. Notably, fish calcium was demonstrated to have a high efficiency of bone mineralization without adverse effects, making it promising as a dietary supplement for bone health [54]. Therefore, several commercial products containing fish calcium have been marketed as dietary or supplement calcium, such as Kalsio [55] and calcium fish bone powder [56]. Although the solubility and bioavailability of fish bone calcium have not yet been compared to those of lobster calcium, this could be iteratively performed based on their nutritional attributes and indicators. The superior solubility of lobster minerals in water (98.4%) combined with the high solubility of its gastric-digested calcium (64%, equivalent to 66.8 mg/g) obtained in this study, compared to the lower solubility (10%) or insolubility at around pH 7 of different fish bone products [50] and 2.88 mg/g of gastric-digested fish bone calcium [57] previously reported, highlights the promising marketability of the lobster mineral.

### 2.4. Nutraceutical Effects of Lobster Calcium on Human Bone and Skin Cells

#### 2.4.1. Lobster Calcium Significantly Stimulated the Proliferation of Human Bone Cells

**Figure 5:**
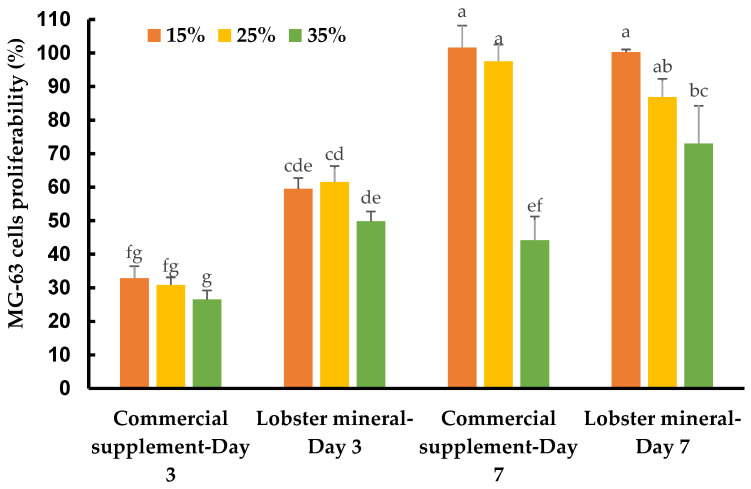
Response and proliferation of MG-63 cells supplemented with lobster minerals and a commercial calcium supplement (CCS) at 15%, 25%, and 35% ratios after three and seven days of incubation. The results are shown as the mean of triplicates with standard deviations. Plots with the same letter(s) are statistically insignificant (*p* > 0.05).

Calcium is an essential nutritional factor for bone formation, growth, and bone health. An adequate amount of nutritional calcium is critical to achieve or maintain optimal bone mass, and thus prevent various bone diseases. Lobster calcium with its higher solubility and good bioavailability may have excellent nutraceutical effects on healthy bone metabolism. This was tested on human osteoblast-like bone cells, which lay down the organic bone matrix in the body. Supplementing lobster calcium to the medium to grow the bone cells, at ratios of 15%, 25%, and 35% (*v*/*v*, equivalent to concentrations of 1237.5, 2062.5, and 2887.5 µg/mL), did not induce any detectable changes in cell morphology and apoptosis. However, it dramatically affected the cell response, growth, and proliferation depending on the mineral product, incubation time, and supplement ratios (Figure 5).

The MG-63 cells responded quickly after three days of incubation. They grew much faster with lobster mineral supplementation at all ratios (15–35%) when compared with CCS (Figure 5 and Table 1). The cell growth rate of the lobster mineral supplement was nearly double that of the CCS (49.9–61.6% vs. 26.5–32.9%). The faster response and proliferation of MG-63 cells supplemented with the lobster mineral could be supported by the favorable nutritional advantages of the lobster product (rich calcium, superior calcium solubility, and high bioavailability) over its commercial counterpart. Notably, the cells proliferated significantly after seven days of incubation to reach 100.3% and 101.6% cell growth at 15% supplementation of the lobster mineral and CCS, respectively. Cell proliferation at the 15% supplementation was significantly higher than that of the 35% supplementation. Although there was an insignificant difference in cell proliferation between the lobster mineral and CCS at 15% supplementation, the cells supplemented with 35% of the lobster mineral were found to grow faster than that with the CCS supplement (73.1 vs. 44.2%). The lower growth rate, in this case, could be influenced by the lack of nutrients from the extra-cellular environment, but still, the significantly higher cell proliferation observed with the lobster mineral supplementation reveals its nutritional advantages over its commercial rival. All the findings indicate that lobster calcium is non-toxic to the osteoblast human bone cells at the investigated concentrations, and it considerably improves bone cell growth, which is positively correlated with healthy bone growth and mineralization.

#### 2.4.2. Lobster Calcium Appreciably Mediated the Growth of Human Skin Cells

**Figure 6:**
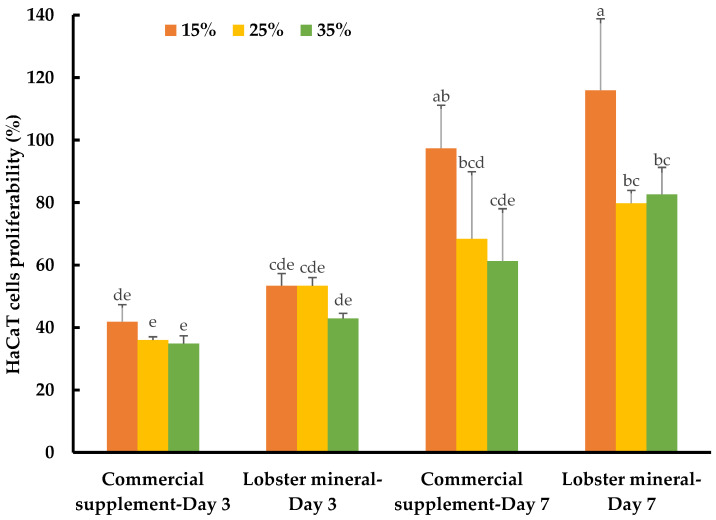
Response and proliferation of HaCaT cells supplemented with lobster mineral and a commercial calcium supplement (CCS) at ratios of 15%, 25%, and 35% after three and seven days of incubation. The results are shown as the mean of triplicates with standard deviations. Plots with the same letter(s) are statistically insignificant (*p* > 0.05).

While calcium is well-known for its vital roles in bone formation and bone health, the biological functions of this nutraceutical element in wound healing and skin health have not yet been thoroughly reported. Recently, the role of calcium in skin regeneration and reconstruction during wound healing has been emphasized [38], while the positive relationship between calcium and healthy skin glow has also been reported [58]. Notably, calcium-based nanoparticles also significantly accelerated skin wound healing [59]. Such studies have inspired our investigation of the lobster mineral for its potential benefits for such cosmeceutical and nutraceutical effects. This was performed by assessing the response and proliferation of human keratinocyte cells (HaCaT) with lobster mineral supplementation in comparison to its CCS rival. Although no detectable changes in cell morphology and apoptosis were found with the supplementation of the two mineral products, the cells grew and proliferated significantly differently according to the products, incubation times, and supplementation ratios (Table 1). The growth rate of HaCaT cells supplemented with lobster mineral at all ratios (15–35%) was found to be as fast as that of its commercial counterpart (cell proliferation of 42.9–53.4% compared to 34.8–41.8% with the CCS supplement) after three days of incubation (Figure 6). The higher solubility and bioavailability of lobster calcium could be responsible for the quick response and high cell growth because this cell type is calcium sensitive [60], while the calcium element was reported to have the ability to activate human skin-derived cells [61]. By day three, an insignificant difference in cell response and growth was found among the three supplemented ratios. However, the growth pattern changed remarkably on day seven. The cells grew much more at the supplementation ratio of 15% compared to 25% and 35% for both lobster mineral (115.9% vs. 79.8–82.6%) and CCS (97.4% vs. 61.3–68.4%). The cells supplemented with the lobster mineral at all ratios after seven days of incubation had a competent or higher cell proliferation than the commercial product. This indicates that lobster calcium is superior in promoting skin cell growth compared to the commercial calcium supplement product. Lobster calcium supplemented at 15% produces the highest cell growth among the three ratios tested, highlighting its commercial applications in wound healing and skin health.

### 2.5. Cytotoxicity of Lobster Mineral Evaluated on Macrophage Cells

**Figure 7:**
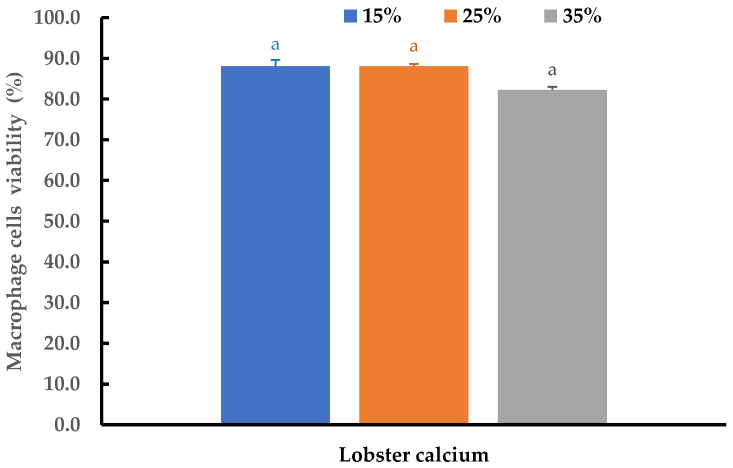
Viability of THP-1 cells after 24 h of exposure to a medium supplemented with lobster minerals at different concentrations for cytotoxicity evaluation. The results are shown as the mean of triplicates with standard deviations. Plots with the same letter(s) are statistically insignificant (*p* > 0.05).

Cytotoxicity and the inflammatory effects of the lobster mineral at different concentrations were further evaluated using THP-1 cell-derived macrophages. At three supplemented ratios of 15%, 25%, and 35%, no changes in morphology were detected, while cell viability at the three tested ratios was insignificantly different (*p* > 0.05). The cell viability ranged from 82.2 to 88.1% (Figure 7). All the viability values are far above the threshold value of 70% as regulated by ISO 10993-5 standards [62]. This result is consistent with the reports from other studies for alternative calcium [51,52,63]. Therefore, lobster minerals could be considered as being safe to be used at the investigated ratios.

## 3. Materials and Methods

### 3.1. Materials

The lobster mineral used in this study was produced by the multi-product biorefinery of lobster shells using microwave-intensified enzymatic deproteinization and lactic-acid demineralization at the optimum condition described in our previous studies [18,20]. The demineralized solution recovered from the process was spray-dried to obtain a white powder product called lobster mineral. The commercial calcium supplement (a calcium complex composed of mainly calcium carbonate and formulated with vitamins D3, K1, and other nutrients, Healthycare) was bought from the Australian Chemist Warehouse in Adelaide. Digestive enzymes and bile salts for gastrointestinal digestion were purchased together with calcium lactate (analytical grade) from Australian Sigma Aldrich. Cells of MG-63 and THP-1 were purchased from ATCC, Manassas, VA, USA, whereas HaCaT was obtained from cell line services, Eppelheim, Germany.

### 3.2. Structural Characterization of Lobster Mineral and Analyses of Its Mineral Profile, Calcium, Solubility, Functionalities, and Bioavailability

#### 3.2.1. Scanning Electron Microscopy

The lobster mineral was sent to Flinders Microscopy and Microanalysis to analyze its crystal structure using the FEI Inspect F50 Scanning Electron Microscopy (FEI Company, Hillsboro, OR, USA). The instrument is equipped with an Energy Dispersive X-Ray (EDX) detector for elemental analysis and an Electron Backscatter Diffraction (EBSD, Concord, MA, USA) detector for crystal analysis. About 2 mg of dry lobster mineral powder was dissolved in MilliQ water and drop-casted on silicon wafers. The samples were then sputtered with a 5 nm platinum coating before they were inserted onto the sample stage. Images were recorded and taken at a voltage of 5 kV and a working distance of 8 mm.

#### 3.2.2. Inductively Coupled Plasma Mass Spectrometry (ICP-MS) Analysis of Lobster Minerals

The lobster mineral profile and its calcium content used for all calculations of this study were analyzed at Flinders Analytical Centre using inductively coupled plasma mass spectrometry (ICP-MS, Santa Clara, CA, USA) as described by Shi, Francis [64]. Briefly, samples (50 mg) were mixed with 3 mL of concentrated nitric acid before incubating overnight and then heated for 1 h (100 °C). After cooling, hydrochloric acid (2 mL) was added and the sample was heated for 1 h (100 °C). Peroxide (1 mL) was added to the cooled samples before they were heated at 100 °C for a further hour, and then these were brought up to 50 mL with MilliQ water for analysis using ICP-MS (Agilent 8900 ICP-QQQ, triple quads, Santa Clara, CA, USA). The system was equipped with an ASXpress plus autosampler with a 1 mL sample loop and Nickel interface cone. It has a standard introduction system composed of a quartz double-pass spray chamber, a quartz torch with a 2.5 mm id injector, and a mictroMist nebulizer (G3266-80005). Calibration standards used for calculations were prepared in the range of 1 ppb–1 ppm.

#### 3.2.3. Water and Oil Binding Capacity of Lobster Mineral

Lobster mineral and its commercial rivals’ ability to influence meat products’ water-binding capacity was quantified by the procedures described by Geirsdottir, Sigurgisladottir [65]. The sample (0.1 g) was added to beef mince (4.9 g) and homogenously mixed to obtain the incorporated beef mince, while the control was solely beef mince (5 g). The beef mince with and without the incorporation was placed in 50-mL centrifuge tubes containing MilliQ (W_W,_ g). They were mixed by vortex for 60 s before leaving to stand on ice for 30 min. The free water was separated by centrifugation at 3000× *g* for 10 min (Beckman Coulter, Allegra X-12R, SN: ALX08813, Brea, CA, USA). The tubes before (WT_B_, g) and after the separation of free water (WT_A_, g) were weighed to compute the water binding capacity of the beef mince.

$$\mathrm{Water}\;\mathrm{binding}\;\mathrm{capacity}\;(\%)=\frac{WT_{B}-WT_{A}}{W_{W}}\;\times \;100$$

The oil binding capacity of lobster mineral was measured according to the method described by Beuchat [66]. A sample with a known weight (W_S_, g) was homogenously vortexed with canola oil (O_A_, mL) in a 50-mL centrifuge tube for 30 s. After the samples were left at room temperature (20 ± 3 °C) for 30 min, they were centrifuged at 5000× *g* for 30 min (Beckman Coulter, Allegra X-12R, SN: ALX08813, Brea, CA, USA). All the tubes were decantated at a 45° angle to separate the free oils (O_F_, mL), and their volumes were measured in a 10-mL graduated cylinder.

$$\mathrm{Oil}\;\mathrm{binding}\;\mathrm{capacity}\;(\mathrm{mL}/g)=\frac{O_{A}-O_{F}}{W_{S}}$$

#### 3.2.4. Solubility of Lobster Minerals and Commercial Calcium Complex

The solubility of lobster mineral and its commercial counterpart was determined following the procedures described by Chaiwanon, Puwastien [53] with slight modifications. Samples with known weights (W_S_) were added to centrifuge tubes containing MilliQ water (40 mL per gram). After solubilizing by vortex for 60 s, the tubes were centrifuged at 14,000 rpm for 10 min to separate the soluble and insoluble fractions. The insoluble residues (R_I_) were collected and dried at 105 °C until a constant weight was achieved. The solubility of the samples (SS) was calculated as follows:

$$\mathrm{SS}\;(\%)=(\frac{W_{S}-R_{I}}{W_{S}})\;\times \;100$$

### 3.3. In Vitro Simulated Gastrointestinal Digestion

The in vitro bioavailability of lobster calcium was determined according to the method of Shen, Luten [67] with minor modifications. This two-part method includes a gastric stage and an intestinal stage as described below.

Gastric stage: Samples (2.5 g) containing a known amount of calcium (Ca_T_) were homogenized with 22.5 mL of MilliQ water. The homogenate was adjusted to pH 2.0 with HCl (6 M) before adding pepsin for pepsin–HCl digestion (0.5 g pepsin per 100 g samples). The mixture was incubated at 37 °C for 2 h in an orbital shaking incubator.

Intestinal stage: Segments of dialysis tube (molecular mass cut-off value 12,000–14,000 Da) containing 25 mL of MilliQ water and an amount of NaHCO_3_ equivalent to the titratable acidity (the combined pepsin digest and pancreatin–bile salts mixture reached pH 7.5) was placed in a 100-mL beaker filled with 20 g of the pepsin digest. The beaker was then incubated in a water bath (37 °C) for 30 min. Next, the pancreatic–bile salt mixture (5 mL) prepared by dissolving pancreatin (3 g) and bile salt (7 g) in one liter of NaHCO_3_ 0.1 M was added and incubated for 2 h. After incubation, the dialysis tubes were removed and rinsed with MilliQ water before determining the volume of their contents (dialysates). The calcium concentration in the dialysates (Ca_B_) and the soluble fraction (Ca_SF_) were determined using the ICP-MS method. The solubility of the calcium in the samples and its in vitro bioavailability were calculated as described in Formulas (1) and (2):

(1) $$\mathrm{Calcium}\;\mathrm{solubility}\;(\%)=\frac{Ca_{B}-Ca_{SF}}{Ca_{T}}\;\times \;100$$

(2) $$\mathrm{Bioavailability}\;(\%)=\frac{Ca_{B}}{Ca_{T}}\;\times \;100$$

### 3.4. In Vitro Effects of Lobster Minerals on Response and Proliferation of Bone and Skin Cells

#### 3.4.1. Human Osteoblast-like Bone Cells (MG-63)

The human osteoblast-like cell line MG-63 (CRL-1427, ATCC, VA, USA) was used to investigate the effect of lobster calcium on bone formation and growth. These cells were grown in growth media (Dulbecco′s Modified Eagle′s Medium, DMEM) containing 10% heat-inactivated fetal bovine serum (FBS, Gibco) supplemented with 1% streptomycin/penicillin (Gibco-BRL, Adelaide, Australia). Lobster calcium powder (330 mg) was solubilized in MilliQ water (40 mL) to obtain a homogenous lobster calcium solution (LCS, pH 7.5) with a concentration of 8.25 mg/mL to be used for testing. CCS (one tablet) was grounded into a fine powder and solubilized in MilliQ water with an amount and volume the same as used for the lobster mineral. The soluble fraction obtained after centrifugation was used for the tests. The cells were loaded with either DMEM only as controls or DMEM supplemented with LCS at 15%, 25%, and 35% (*w*/*v*) of the well (100 μL/well) in 96-well tissue culture plates (flat bottom) at a density of 10^5^ cells/mL. The plates were then incubated for three and seven days at 37 °C in a moistened chamber with 5% CO_2_. After three and seven days of incubation, the cells were observed under microscopy to assess their morphology. Cell viability and proliferation were evaluated with the methyl-thiazolyl tetrazolium (MTT) assay. Typically, 10 μL of MTT solution (5 mg/mL in FBS) with 90 μL fresh medium was added to each well containing cells at a density of 10^5^ cells. Then, the plates were incubated in the dark at 37 °C under 5% CO_2_ for 3–4 h until there was a noticeable formation of blue/purple crystals. The yellow soluble MTT solution was reduced to a blue/purple amethyst formazan crystal by the mitochondria of viable cells. Thus, the more blue/purple crystals were produced, the more viable cells were presented. The formazan crystals at the bottom of the wells were retained by carefully aspirating the cell culture media from each well. Then 100 μL of dimethyl sulfoxide was added to each well with gentle shaking to solubilize the formazan crystals by leaving the plates at room temperature for 30 min. The optical density of the color solutions (purple) was measured at 570 nm using a multi-well plate reader (SYNERGY-HTX, Bio-Tek, Waltham, MA, USA). The number of viable osteoblast cells is proportional to the obtained optical density value. Results were normalized to the tissue culture plate (TCP, a control).

#### 3.4.2. Human Keratinocytes (HaCaT Cells)

Since calcium has been reported to play several vital functions in wound healing, keratinocyte cells derived from adult human skin, HaCaT (300493, cell line services, Eppelheim, Germany), were used to investigate skin cell response and proliferation after supplementation with the lobster calcium solution at ratios of 15%, 25%, and 35% (*w*/*v*). The cell morphology was evaluated under microscopy while their viability or proliferation was assessed using the methyl-thiazolyl tetrazolium (MTT) assay with protocols as previously described in the 3.4.1 subsection. The color of the solutions (purple) obtained at the final step was measured at 570 nm, and their optical density was normalized to the TCP.

### 3.5. Cytotoxicity of the Lobster Mineral Evaluated on Macrophage Cells (THP-1)

The in vitro cytotoxicity of lobster mineral was tested on macrophages derived from THP-1 cells (TIB-202, ATCC, VA, USA). These cells were maintained in RPMI 1640 (Gibco) supplemented with 10% heat-inactivated fetal bovine serum (Gibco-BRL) and 1% streptomycin/penicillin (Gibco-BRL) at 37 °C, 5% CO_2_ in an incubator (Sanyo MIR-162, Osaka, Japan). Briefly, THP-1 cells were cultured in 75-cm^2^ culture flasks (Corning) with the culture medium containing 0.1 µg/mL phorbol 12-myristate-13-acetate (PMA) (Gibco-BRL). They were incubated for 2 days at 37 °C in a humidified atmosphere of 5% CO_2_ to allow them to differentiate into macrophages. Once reaching about 80–85% confluence, they were harvested using 0.25% Trypsin-EDTA (1X) (Gibco-BRL). The macrophages were then seeded at 10^5^ cells/well density in the flat-bottomed 96-well plates (Costar). The seeded-cell wells were then supplemented with the media formulated with different volumes of lobster shell calcium solutions to reach the final ratios of 15%, 25%, and 35%, corresponding to concentrations of 1237.5, 2062.5, and 2887.5 µg/mL, respectively. The exposure of these macrophage cells to lobster mineral occurred while the plates were incubated at 37 °C in a moistened chamber with 5% CO_2_ for 24 h. After the exposure time, the cells were washed three times with Phosphate Buffer Saline (PBS) (pH 7.3). The aged media was then removed before 10 μL MTT (0.5 mg/mL) and 90 μL fresh media were added to each well and further incubated at 37 °C for 4 h. Since only the viable cells have functional mitochondrial dehydrogenase enzymes which can reduce MTT to formazan, the viability of the macrophages was determined with an MTT assay as performed in Section 3.4. The cytotoxicity of lobster mineral was assessed according to the cell viability based on the ISO 10993:5 standard as described in the study of Leslie, Vasanthi Bathrinarayanan [62].

### 3.6. Statistical Analysis

All observations in this study were performed in triplicate. The obtained data were subjected to an analysis of variance using R studio version 1.3.1093 (2009–2020 RStudio, PBC, Boston, MA, USA) with the support of several packages such as dplyr, multcompView, and DoE. The Tukey test was used for conducting multiple comparisons and post-hoc tests to determine significant differences among the means (*p* < 0.05).

## 4. Conclusions

Calcium in the form of lobster mineral recovered from underutilized lobster shells in a multi-product biorefinery using microwave intensification combined with lactic acid for demineralization has a favorable calcium ratio and nutritional profile. More importantly, lobster mineral has better solubility and bioavailability as well as water and oil bind capacities when compared to commercial rivals (CCS, CCL, and casein). The in vitro tests of the lobster mineral demonstrated its superior ability in promoting the cell proliferation of both human bone and skin cells, showing non-appreciable cytotoxicity in macrophage cells. These results provide further evidence to support lobster mineral as a promising and commercial alternative calcium supplement for bone and skin health. Further studies in vivo and clinical trials are also warranted to provide concrete evidence for the commercial application of lobster minerals in multiple calcium-based health and nutraceutical products.

## Figures and Tables

**Table 1:** Summary variant analysis of the effects of different mineral products, incubated time, and supplemented ratios on the viability of MG63 and HaCaT cells.

Source	DOF ^1^	MG-63 Cells				HaCaT Cells			
		SS ^2^	MS ^3^	FV ^4^	pV ^5^	SS	MS	FV	pV
Model	11	25,614.19	2328.56	82.49	<0.0001 *	21,675.77	1970.52	18.07	<0.0001 *
A-Mineral product	1	2366.82	2366.82	83.85	<0.0001 *	1941.87	1941.87	17.81	0.0003 *
B-Incubated time	1	14,661.17	14,661.17	519.39	<0.0001 *	14,762.25	14,762.25	135.41	<0.0001 *
C-Supplemented ratio	2	4344.90	2172.45	76.96	<0.0001 *	3212.15	1606.08	14.73	<0.0001 *
AB	1	1014.42	1014.42	35.94	<0.0001 *	51.36	51.36	0.47	0.4991
AC	2	450.91	225.45	7.99	0.0022 *	0.77	0.39	0.00353	0.9965
BC	2	1909.61	954.80	33.83	<0.0001 *	1563.25	781.63	7.17	0.0036 *
ABC	2	866.35	433.18	15.35	<0.0001 *	144.11	72.06	0.66	0.5255
Pure error	24	677.46	28.23			2616.47	109.02		
Cor total	35	26,291.65				24,292.24			

^1^ Degree of freedom, ^2^ Sum of the square, ^3^ Mean square, ^4^ F-value, ^5^ *p*-value, * Significant with *p* < 0.05.

## Data Availability

Not applicable.
